# Supplementary material for: Defense against phytopathogens relies on efficient antimicrobial protein secretion mediated by the microtubule-binding protein TGNap1
Source: Nat Commun. 2023 Oct 11;14:6357. doi: 10.1038/s41467-023-41807-4 (PMC10567756; doi:10.1038/s41467-023-41807-4)
Supplement: Supplementary file 1 — Supplementary Information [file 41467_2023_41807_MOESM1_ESM.pdf]

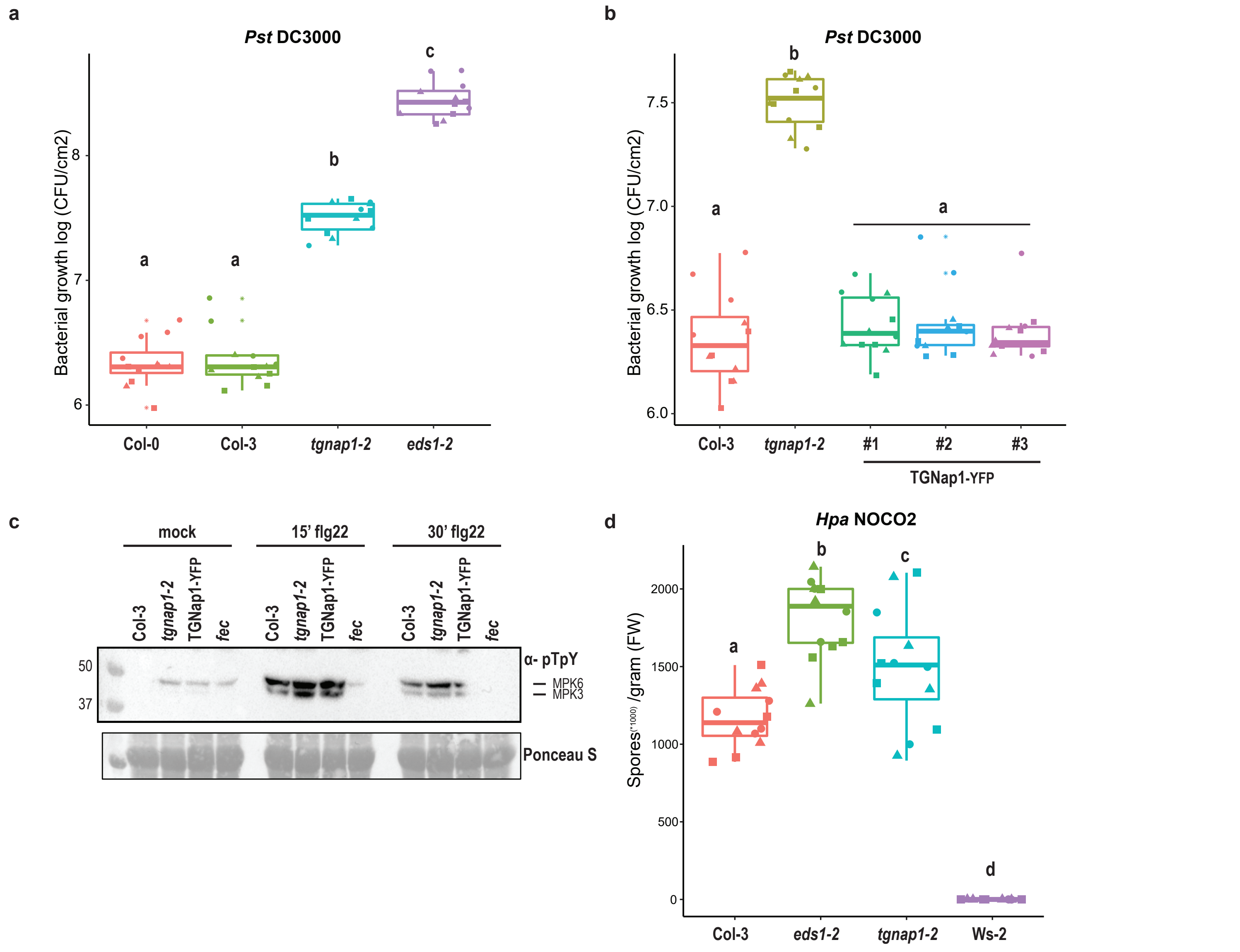

Fig. S1 The susceptibility of *tgnap1-2* to *Pst* DC3000 is specifically linked to TGNap1

(a) Four-week-old *Arabidopsis* plants of the indicated genotypes were infiltrated with *Pst* DC3000. Bacterial titers were determined at 3 dpi. Data from three independent experiments are represented in a box plot with data points. Individual data points are represented with different shapes. Statistical differences between genotypes were analysed using ANOVA (Tukey's HSD, $p < 0.05$).

(b) Four-week-old *Arabidopsis* plants of the indicated genotypes and three independent stably transformed TGNap1-YFP complemented lines were infiltrated with *Pst* DC3000. Bacterial titers were determined at 3 dpi. Data from three independent experiments are represented in a box plot with data points. Individual data points are represented with different shapes. Statistical differences between genotypes were analysed using ANOVA (Tukey's HSD, $p < 0.05$).

(c) Phosphorylation levels of MPK3 and MPK6 upon mock and flg22 treatment were monitored by Western blotting with anti-pTpY serum. Protein loading was monitored by Ponceau S.

(d) Quantification of *Hpa* NOCO2 spores at 5 dpi. Data from three independent experiments are represented in a box plot with data points. Individual data points are represented with different shapes. Statistical differences between genotypes were analysed using ANOVA (Tukey's HSD, $p < 0.05$).

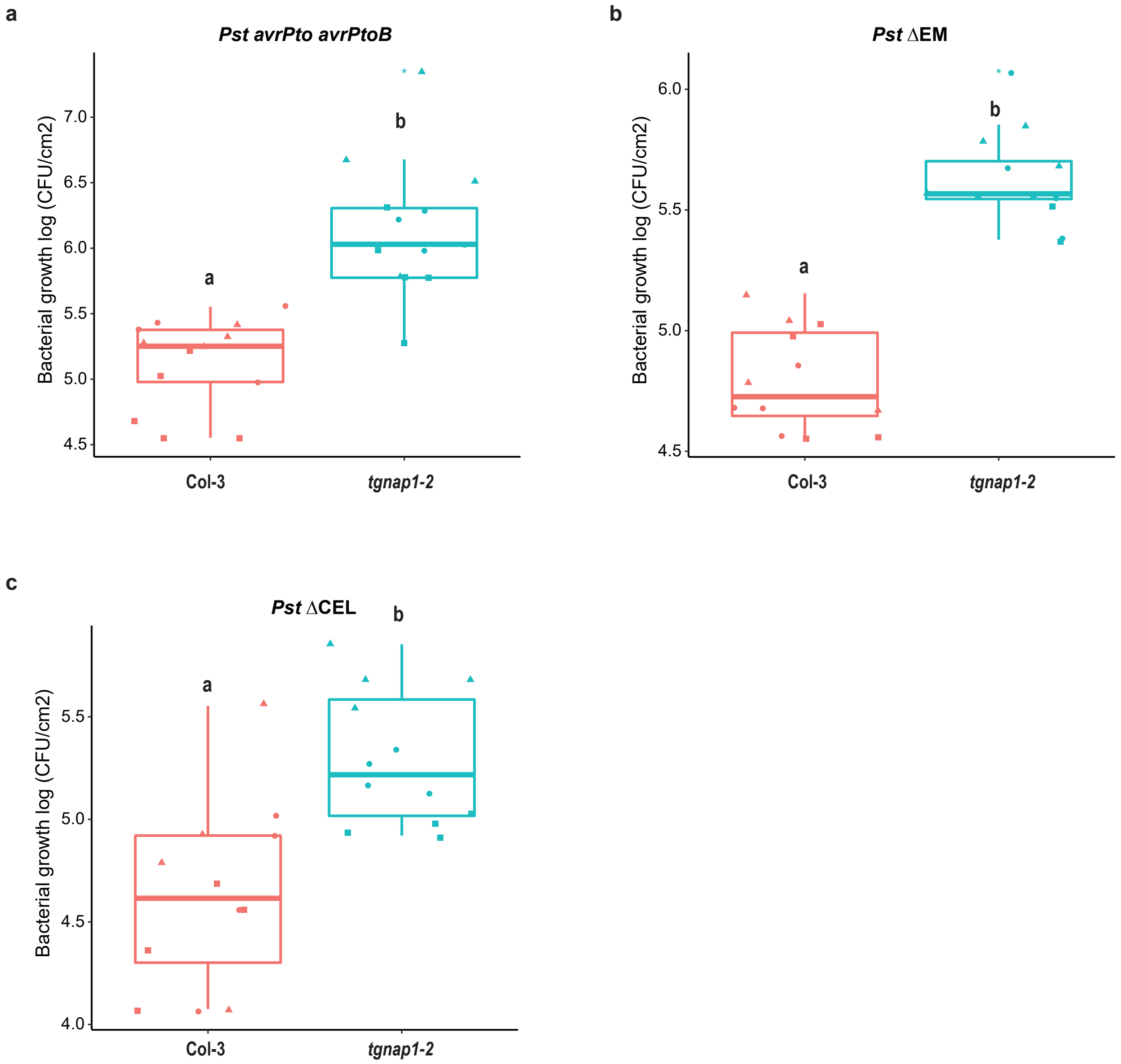

Fig.S2 TGNap1's role in immunity is not restricted to a limited Pst effector range

Four-week-old *Arabidopsis* plants of the indicated genotypes were infiltrated with **(a)** *Pst avrPto avrPtoB*, **(b)** *Pst* ΔEM, **(c)** *Pst* ΔCEL. Bacterial titers were determined at 3 dpi. Data from atleast three independent experiments are represented in a box plot with data points. Individual data points are represented with different shapes. Statistical differences between genotypes were analysed using ANOVA (Tukey's HSD, $p < 0.05$).

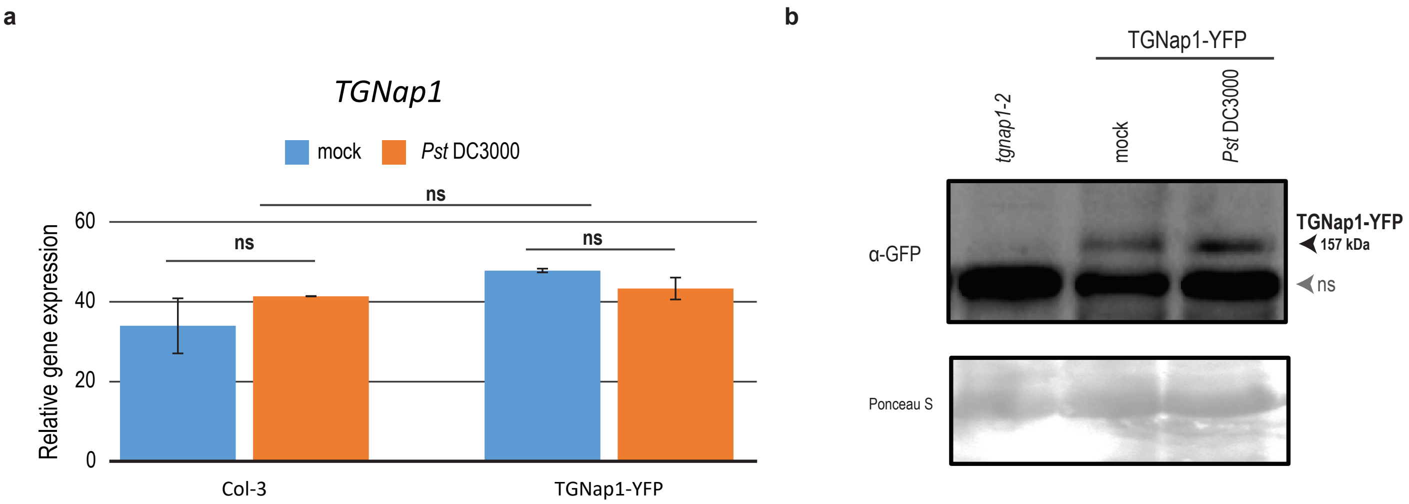

Fig. S3 TGNap1 protein accumulation does not change upon infection with *Pst* DC3000
(a) Gene expression of *TGNap1* was analyzed with qRT-PCR. Values are represented as means ± S. E. Experiments were repeated twice with similar results. ns - no siginificant difference.
(b) Accumulation of TGNap1-YFP in four-week-old leaves of TGNap1-YFP transgenic plants treated with mock or *Pst* DC3000 probed with α-GFP serum. *Tgnap1-2* leaves were used as control to ascertain specificity of protein bands. A non-specific (ns) band observed is marked with a grey arrow. Ponceau S was used as loading control.

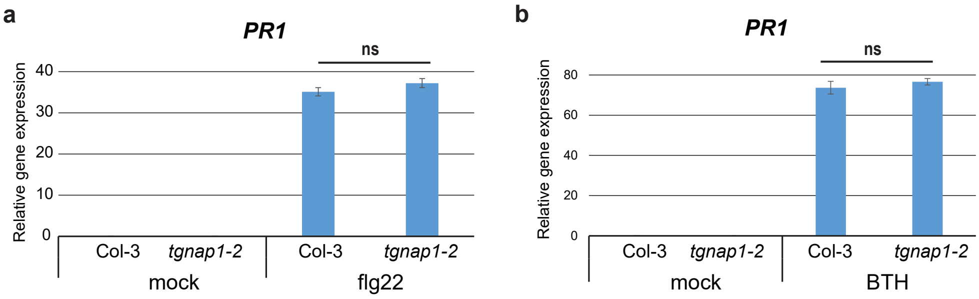

Fig. S4 ***PR1*** **expression upon flg22 and BTH treatment**
Four-week-old plants were infiltrated with **(a)** flg22 or **(b)** BTH. Gene expression of *PR1* was analyzed with qRT-PCR. Values are represented as means ± S.E. Experiments were repeated two times. ns **-** no siginificant difference.

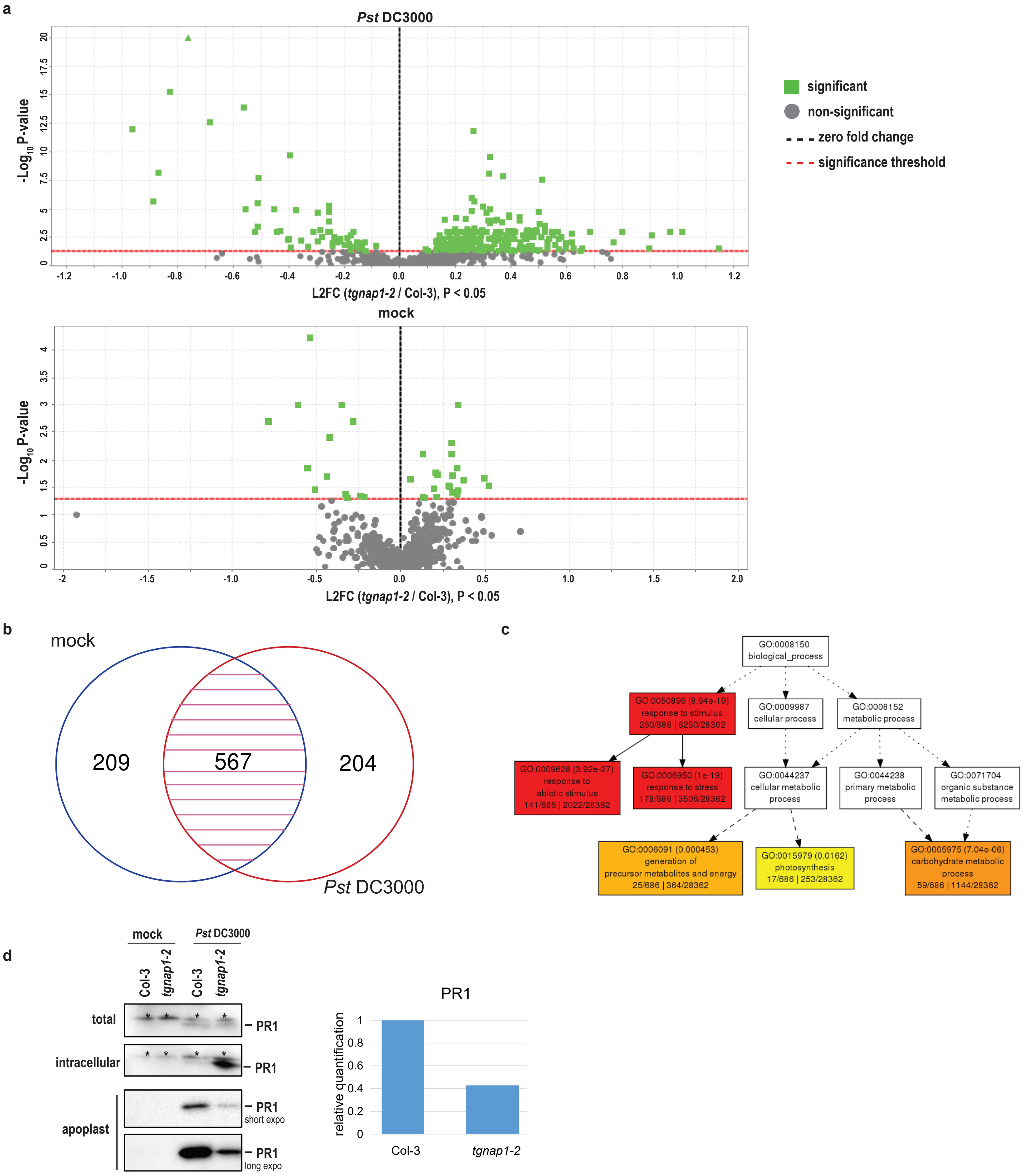

Fig. S5 Apoplastic proteins identified in *tgnap1-2* upon *Pst* DC3000 infection

(a) Leaf apoplastic fluid was isolated 24 hpi with either mock or *Pst* DC3000. Isolated apoplastic fluid were subjected to label-free LC-MS analysis. Proteins detected with at least 2 peptides with a 1%FDR were selected for analysis. Proteins were filtered using a permutation test at p <0.05 (without multiple testing).

(b) Venn diagram of proteins identified in the apoplastic proteome of mock and *Pst* DC3000 treated Col-3 and *tgnap1-2* plants.

(c) Gene ontology enrichment of 567 overlapping proteins found in mock and *Pst* DC3000 treated Col-3 and *tgnap1-2* plants.

(d) Leaf apoplastic fluid probed with α-PR1 after treatment with either mock or *Pst* DC3000.

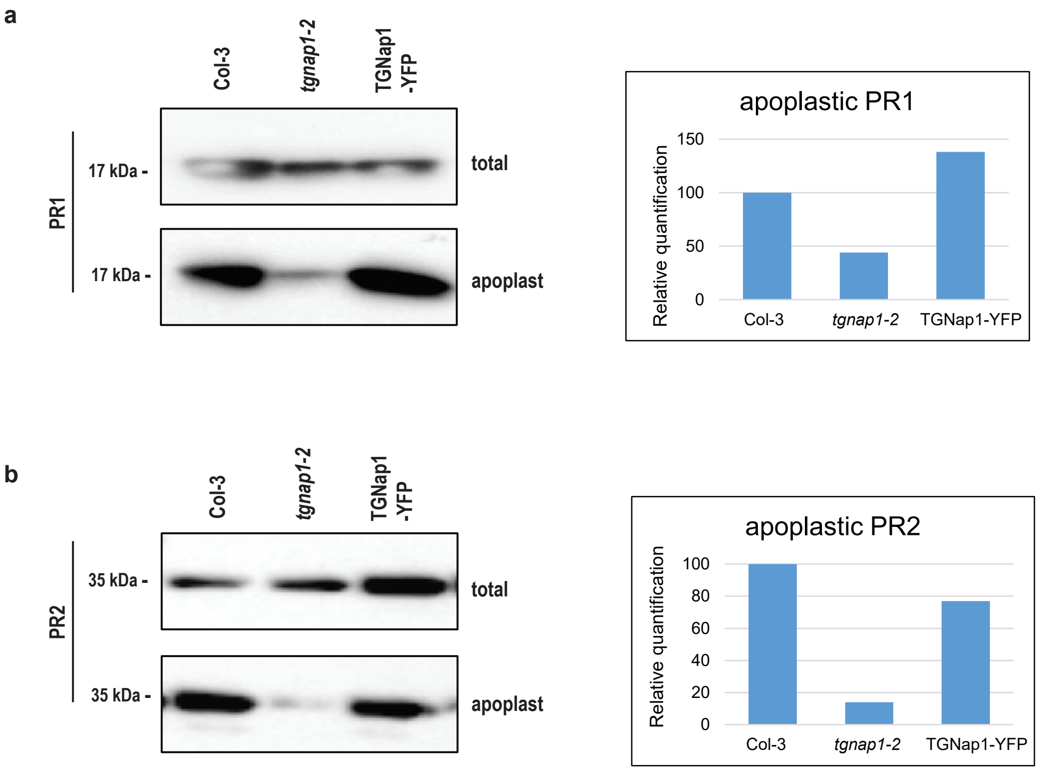

Fig. S6 Apoplastic protein levels of PR1 and PR2 upon *Pst* DC3000 infection

Leaf apoplastic fluids probed with **(a)** α-PR1 or (b) α-PR2 after *Pst* DC3000 treatment. Relative quantification of band intensity normalized to Col-3.

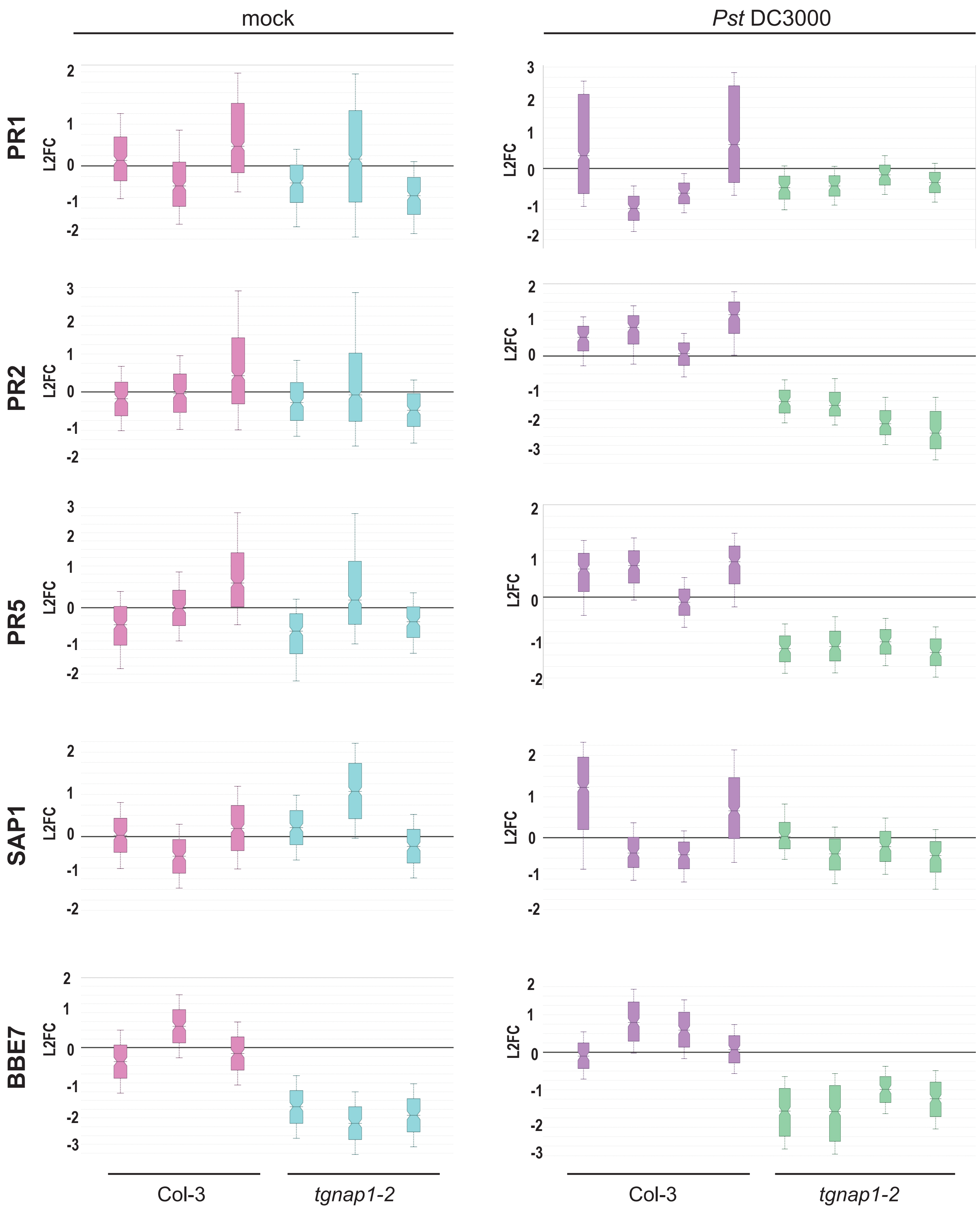

Fig. S7 Protein levels detected in the apoplast of mock and *Pst* DC3000 leaves
Protein levels of indicated proteins represented with log2 fold-change (L2FC) values. Individual replicates represented as box plots.

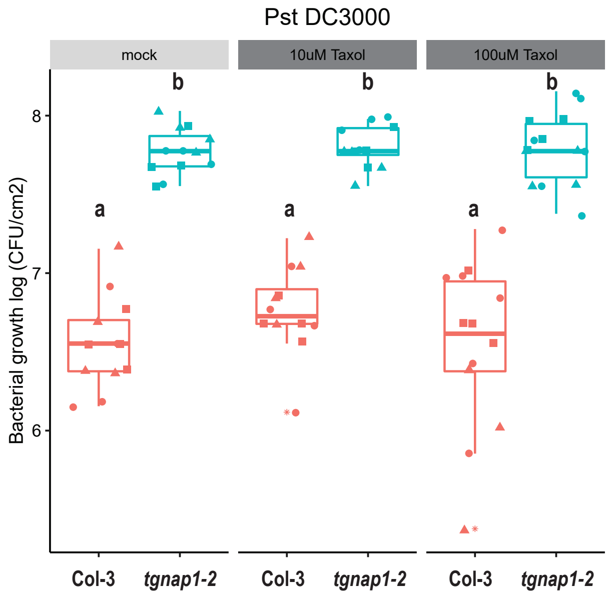

Fig. S8 Microtubule stabilization does not compensate for loss of TGNap1
Four-week-old *Arabidopsis* plants of the indicated genotypes were infiltrated with *Pst* DC3000 supplemented with either 10mM $MgCl_2$ (mock) or taxol at the indicated concentrations. Bacterial titers were determined at 3 dpi. Data from at least three independent experiments are represented in a box plot with data points. Individual data points are represented with different shapes. Statistical differences between genotypes were analysed using ANOVA (Tukey’s HSD, $p < 0.05$).

Primer	Sequence	Purpose
TGNap1_F	ccacaaagtttgttggaggag	genotyping *tgnap1-2*
TGNap1_R	tcagaaagaacatacaaagccg	
SAIL_insertion	gcttcctattatatcttcccaaattaccaataca	
qGapDH_F	ccatcaaggaccctcagtcc	qRT-PCR
qGapDH_R	tgccttcagattcctccctg	
qUBQ10_F	ggtttgtgttttggggccttg	
qUBQ10_R	cgaagcgatgataaagaagaagttcg	
qTGNap1_F	ttcctcacacgacagaacgg	
qTGNap1_R	gtgcacgaatggcttcacag	
qICS1_F	tactaaccagtccgaaagacg	
qICS1_R	gaggcttgacaacaactctgt	
qPR1_F	ttcttccctcgaaagctcaa	
qPR1_R	aaggcccaccagagtgtatg	
TGNap1_1-300_F	cttaaaatgcatatttaagggtctcgcgctg	
TGNap1_1-300_R	cagcgcgagacccttaaatatgcattttaag	
sid2-2_A	ttcttcatgcaggggaggag	genotyping *sid2-2*
sid2-2_B	gcaccagcttttatcggaaa	
sid2-2_C	cacaaacagctggagttgga	
min7_LP	ctacatttgctccctctgtgc	genotyping *min7*
min7_RP	ttcttctctgctgtcaggctc	

Table S1: Primers used in the study
