# Supplementary material for: Defense against phytopathogens relies on efficient antimicrobial protein secretion mediated by the microtubule-binding protein TGNap1
Source: Nat Commun. 2023 Oct 11;14:6357. doi: 10.1038/s41467-023-41807-4 (PMC10567756; doi:10.1038/s41467-023-41807-4)
Supplement: Supplementary file 3 — Description of Additional Supplementary Files [file 41467_2023_41807_MOESM3_ESM.pdf]

Description of Additional Supplementary Files

Supplementary Data 1: List of DEGs upon *Pst* DC3000 treatment compared to mock in Col-3 and *tgnap1-2*.

Supplementary Data 2: List of proteins detected in the apoplast of Col-3 and *tgnap1-2* upon *Pst* DC3000 and mock-treated samples. Selection cut-off: A minimum of 2 peptides with a 1% false discovery rate.

Supplementary Data 3: Gene ontology terms associated with proteins found in the apoplast of mock and *Pst* DC3000 treated samples.

Supplementary Data 4: List of differentially abundant proteins (±1 fold-change, $p < 0.05$ permutation test: Benjamini-Hochberg) detected in the apoplast of Col-3 and *tgnap1-2* upon *Pst* DC3000 and mock treatments. Selection cut-off: A minimum of 2 peptides with a 1% false discovery rate.
